# Challenges and lessons learned from using anchoring vignettes to explore quality of life response behavior

**DOI:** 10.1007/s11136-020-02488-4

**Published:** 2020-04-18

**Authors:** Janine Topp, Christoph Heesen, Matthias Augustin, Valerie Andrees, Christine Blome

**Affiliations:** 1 Institute for Health Services Research in Dermatology and Nursing (IVDP), University Medical Center Hamburg-Eppendorf (UKE), Martinistraße 52, Hamburg, 20246 Germany; 2 Institute of Neuroimmunology and Multiple Sclerosis (INIMS), University Medical Center Hamburg-Eppendorf (UKE), Martinistraße 52, Hamburg, 20246 Germany; 3 Department of Neurology, University Medical Center Hamburg-Eppendorf (UKE), Martinistraße 52, Hamburg, 20246 Germany

**Keywords:** Anchoring vignettes, Health-related quality of life, SF-12, Response shift, Mixed-methods

## Abstract

**Purpose:**

Asking patients to rate health-related quality of life (HRQoL) of hypothetical individuals described in anchoring vignettes has been proposed to enhance knowledge on how patients understand and respond to HRQoL questionnaires. In this article, we describe the development of anchoring vignettes and explore their utility for measuring response shift in patients’ self-reports of HRQoL.

**Methods:**

We conducted an explorative mixed-methods study. One hundred patients with multiple sclerosis or psoriasis participated in two interviews at intervals of 3–6 months. During both interviews, patients assessed HRQoL of 16 hypothetical individuals on the SF-12 questionnaire (two vignettes for each of the eight domains of the SF-12). In addition to these quantitative ratings, we used the think-aloud method to explore changes in patients’ verbalization of their decision processes during vignette ratings.

**Results:**

Agreement of vignette ratings at baseline and follow-up was low (ICCs < 0.55). In addition, paired sample *t*-tests revealed no significant directional mean changes in vignette ratings. Thus, ratings changed non-directionally, neither confirming retest reliability nor a systematic change of assessment. Furthermore, patients’ verbalization of their decision processes did not indicate whether or not the assessment strategy of individual patients had changed.

**Conclusions:**

Patients’ ratings of anchoring vignettes fluctuate non-directionally over time. The think-aloud method appears not to be informative in exploring whether these fluctuations are due to changes in the individual decision process. Overall, vignettes might not be an appropriate approach to explore response shift, at least with regard to the specific target population and the use of the SF-12.

**Electronic supplementary material:**

The online version of this article (10.1007/s11136-020-02488-4) contains supplementary material, which is available to authorized users.

## Introduction

In health care, we use standardized questionnaires to convert patients’ perceived state of health-related quality of life (HRQoL) into a numerical score. What sounds very simple is indeed a highly complex process. As a basis for completion, patients need to comprehend instructions and questions on various aspects of HRQoL, retrieve relevant memories on their HRQoL regarding a certain time span, make a judgment and map this judgment on the given response scale [1]. This complex process is partly unconscious, not directly observable and might differ intra- and inter-individually: Differences in the interpretation and evaluation of HRQoL hamper comparability between groups of patients as well as comparability of individual HRQoL states over time [2–4]. Therefore, interpretation of HRQoL scores is challenging.

At the same time, HRQoL reports are of high value in research and clinical practice. They are essential for comparing different patient groups as well as for evaluating changes in HRQoL over time. Changes in HRQoL are an indicator of treatment benefit and can support individual decision-making in clinical practice. Although HRQoL reports are not designed to capture objective health, they are supposed to reflect individual perceptions in a way that allows for intra-individual comparisons. This presupposes that HRQoL is interpreted similarly over a disease trajectory. In reality, however, the meaning of one’s self-evaluation may change. This phenomenon is called response shift [5]. Response shift includes three different sub-phenomena that may lead to changes in the measured HRQoL state with no actual changes having occurred: (1) a shift in the individual definition or interpretation of the HRQoL construct (reconceptionalization), (2) a shift in the values that people assign to different domains of HRQoL (repriorization) and (3) a shift in the internal standards of interpreting the measurement tool (recalibration) [6]. It is contestable whether these three sub-phenomena are biases per se or may be a desired adaptation in the course of a disease [7–10]. Incontestable, however, is that it should be attempted to disentangle changes caused by response shift from actual changes in HRQoL.

A promising approach to account for response shift could be the use of anchoring vignettes. Anchoring vignettes are descriptions of hypothetical individuals regarding a particular construct of interest, e.g., HRQoL [11]. Respondents rate these hypothetical individuals on the same scale they use for their self-rating. The vignette ratings add an individual reference frame to the subjective self-rating [2]. Applied longitudinally, this method may provide insight into the response shift phenomenon.

So far, anchoring vignettes have mainly been applied in cross-sectional studies to improve inter-group comparisons of health states [12–14], life satisfaction [15] and HRQoL [16]. In contrast, they have gained little attention in the identification of response shift in longitudinal studies. While Korfage and colleagues considered anchoring vignettes as a useful tool to identify response shift in a longitudinal study [17], Hinz and colleagues concluded that anchoring vignettes are inappropriate to correct self-ratings for individual reference frame and thus to identify response shift [18]. Both articles explored response shift with regard to a single-item visual analogue scale (VAS). To our knowledge, the vignette approach has not been used for multi-item HRQoL questionnaires.

One frequently used, standardized and multi-item HRQoL questionnaire is the Short Form 12 (SF-12), a brief version of the Short Form 36 (SF-36) [19, 20]. Both measures are widely used and well accepted in research and clinical practice. However, statistical approaches indicate that patients’ choice of a response option in the SF-12 and SF-36 can be affected by specific patient characteristics beyond their degree of HRQoL (differential item functioning) [21–23]. As some patient characteristics such as age and health state change over time, the reference frame of the individual patient may also change and a response shift might be present.

This study was originally designed to investigate response shift in the assessment of HRQoL (SF-12) by using anchoring vignettes. In this context, we evaluated the appropriateness of the anchoring vignette approach and faced several challenges throughout the study. This led to the conclusion that the approach might be limited with regard to the initial aim of exploring response shift. As we are convinced that knowing about these challenges would be of high value for researchers, we will outline our lessons learned from applying anchoring vignettes in the context of HRQoL assessment below.

In this study, we focused on patients diagnosed with psoriasis or multiple sclerosis (MS). Both are chronic diseases being associated with significant impairments in HRQoL [24], which emphasizes the need for accurate monitoring of HRQoL.

## Study overview

We developed anchoring vignettes and conducted an exploratory mixed-methods study in which these vignettes were used to explore response shift in the assessment of HRQoL with the SF-12. In the following, we decided to deviate from the classical structure of a scientific article to better delineate the process of method development and continuous evaluation.

Firstly, we outline the development and evaluation of the anchoring vignettes (see Part I). Positive evaluation of anchoring vignettes was a prerequisite for conducting the exploratory mixed-methods study.

**Fig. 1:**
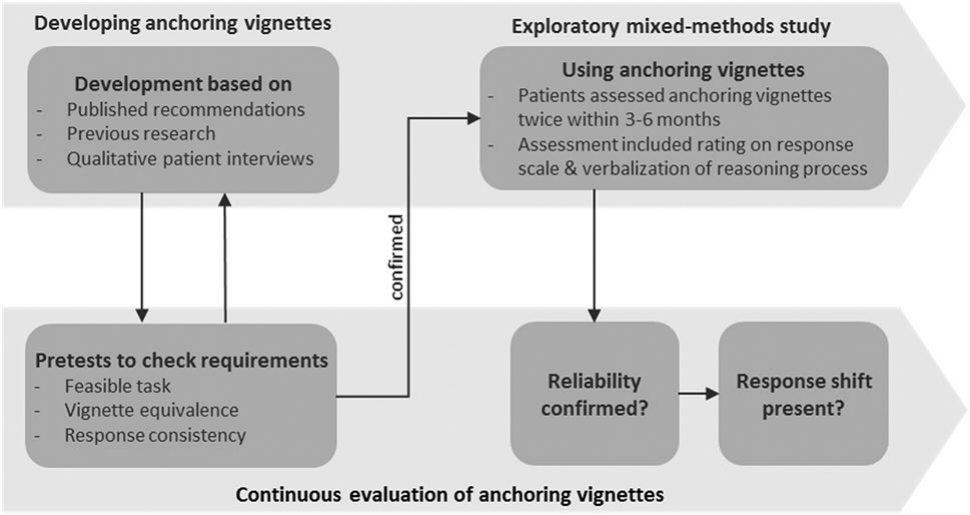
Flow chart on development and continuous evaluation of the anchoring vignette approach

Secondly, we describe how anchoring vignettes (Online Supplementary 1) were used in a patient sample and address challenges and lessons learned regarding the anchoring vignette approach (see Part II). Quantitative and qualitative methods and results are presented (Fig. 1).

## Part I—Developing and evaluating anchoring vignettes

We developed anchoring vignettes by following a multi-stage process that incorporated a literature review and patient interviews. We conducted pretests in a convenience sample (seven healthy individuals, ten patients). As described in detail below, the development resulted in a positive evaluation of the anchoring vignette approach, providing a solid basis for the use of the method in the exploratory mixed-methods study. The evaluation process took into account requirements that must be met in order to draw conclusions on the individual reference frame of patients [2, 14, 25, 26].

A first requirement was to develop a task that is feasible and manageable for study participants. The first approach that we tested was to use comprehensive anchoring vignettes describing hypothetical patients with regard to all eight domains of the SF-12 questionnaire. Pretests revealed that these vignettes were perceived as too long and complex. Participants reported that they had difficulties in extracting relevant information to answer specific questions of the SF-12. Additionally, they stated that they had difficulties in remembering specific characteristics of the hypothetical patient leading to uncertainty in answering some questions. That is why we instead developed separate anchoring vignettes for each of the eight domains of the questionnaire. Healthy individuals and patients stated that the domain-specific anchoring vignettes contained all information necessary to answer the domain-related questions. Furthermore, the overall task was generally feasible and required an adequate level of concentration. A drawback of using domain-specific anchoring vignettes (different vignettes for different domains) is that they cannot be analyzed on a total score level, but on item and domain level only.

As a second requirement, the assumption of response consistency should be met. Response consistency means that when rating anchoring vignettes, patients will use the same standards as they do when rating their own HRQoL [2]. To ensure response consistency, we followed recommendations in the literature and previous research results [2, 14, 25, 27]: study participants were asked to use the same standards for vignette ratings and self-ratings, vignette descriptions did not contain information on age, and vignette descriptions had the same sex and diagnosis as the participant. Additionally, we conducted nine patient interviews to learn about common HRQoL impairments of patients with psoriasis or MS to include those in the vignette descriptions. Direct probing during the pretests revealed that most participants could identify with the anchoring vignettes. They imagined themselves being in the situation of the hypothetical patient or imagined how this patient would have assessed him/herself. However, further analyses of these interviews revealed that participants were less likely to empathize with the hypothetical patient and not use the response categories in the same way in case they had never experienced the stated impairments themselves.

As a third requirement, the assumption of vignette equivalence should be met. Vignette equivalence means that different patients understand the descriptions of HRQoL impairments within the vignettes in the same way; only the choice of response categories is allowed to differ [2]. In accordance with recommendations for achieving vignette equivalence, descriptions were formulated as precisely as possible. Depending on the domain of the questionnaire, each impairment was specified regarding its duration (e.g., “3 days within 4 weeks”) and its extent. The latter was achieved by avoiding vague descriptions (e.g., “major”, “severe”, or “mild” impairment) but instead specifying the impact on daily life. Healthy individuals and patients in the pretests assessed the final anchoring vignettes as being very clear and explicitly phrased.

## Part II—Using anchoring vignettes in a mixed-methods study

After overall positive evaluation of the anchoring vignettes in the pretest, we conducted a longitudinal mixed-methods study to explore response shift. We aimed to recruit 50 patients with psoriasis and 50 patients with MS. Recruitment took place at the psoriasis and MS outpatient clinics of the University Medical Center Hamburg-Eppendorf (UKE). Patients were eligible to participate if they were diagnosed with psoriasis or MS, if they were at least 18 years of age and if the attending physician expected a change in the patient’s health state in the course of the following 3–6 months (e.g., expected change in health state due a change in the medication plan or due to a new diagnosis of MS or psoriasis). The latter inclusion criterion was chosen as response shift is likely to occur after a change in the health state [28]. With this criterion being met, we expected response shift in the study population. Patients who had insufficient cognitive ability to assess anchoring vignettes were excluded.

**Fig. 2:**
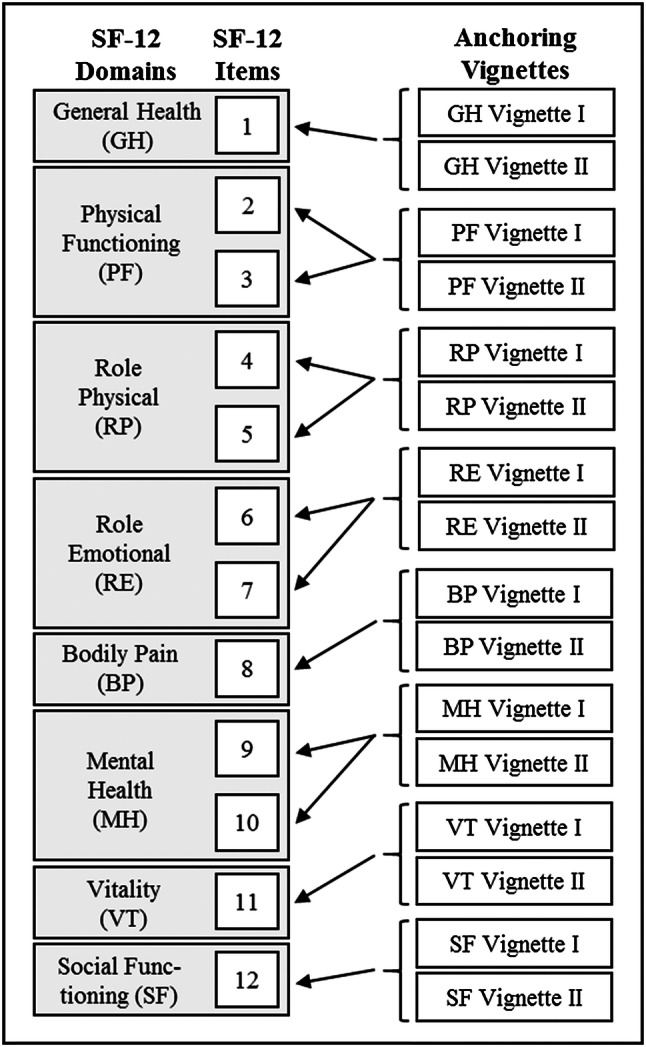
Graphical representation of which anchoring vignette the patients assessed on which domain of the SF-12

Patients participated in two semi-structured, guideline-based interviews: baseline (*t*1) and 3–6 months later (*t*2). Interviews were conducted between July 2017 and September 2018. At both time points, participants assessed their own HRQoL on the SF-12. In a second step, they assessed the HRQoL of hypothetical patients described in anchoring vignettes. Two anchoring vignettes for each of the eight domains were subsequently presented to the participant. Participants rated each vignette with regard to the domain-specific items of the SF-12 (Fig. 2). The think-aloud method [29] was used to gain insight into the individual decision process for each vignette rating. Furthermore, participants completed a questionnaire on sociodemographic characteristics at the end of the first interview. All interviews were audio-recorded.

## Quantitative analysis: methods

### Assumptions

The quantitative analysis of vignette ratings based on the following assumptions:

- Mean changes in the rating of identical anchoring vignettes from *t*1 to *t*2 express changes in the reference frame of the aggregated sample. Changes in the reference frame are supposed to be present if ratings of identical vignettes differ significantly over time according to a paired sample *t*-test.
- Stable ratings of identical anchoring vignettes over time express a stable reference frame, i.e., no response shift. No response shift is supposed to be present if within-person agreement of ratings of identical vignettes is high according to the intra-class correlation coefficient (ICC) for single measures. High agreement indicates a good (test–retest) reliability of the anchoring vignette approach.
- No significant mean change in vignette ratings of the aggregated sample (paired sample *t*-test) and at the same time low within-person agreement of anchoring vignette ratings (ICC) indicate that vignette ratings differ non-directionally. Non-directional fluctuations would mean that (test–retest) reliability of the anchoring vignette approach cannot be confirmed.

### Statistical analysis

Sociodemographic characteristics were summarized using descriptive statistics. SF-12 data of participants were analyzed based on the QualityMetric Inc. manual and 1998 normative data of the U.S. general population [30]. The SF-12 consists of twelve items with three to five response options each. Eight domain scores can be calculated by adding up item responses of a domain to a raw scale score and transforming it to a 0–100 scale score. Domain scores can further be transferred to two norm-based summary scores: a mental component summary (MCS) and a physical component summary (PCS). Higher domain or summary scores indicate better HRQoL.

Concerning the self-ratings at *t*1 and *t*2, MCS and PCS were computed. Concerning the vignette ratings, SF-12 data were analyzed on item and domain level. On item level, changes in vignette ratings were inspected graphically (Online Supplementary 2) and Cohen’s kappa (*κ*) was calculated to determine agreement of responses between *t*1 and *t*2. Furthermore, we calculated SF-12 domain scores which were used to explore above mentioned assumptions: Paired sample *t*-tests investigated whether identical anchoring vignettes were rated systematically different on group level. ICC provided insight into the within-person agreement of anchoring vignette ratings over time. Values of < 0.40, 0.40–0.59, 0.60–0.74 and 0.75–1.00 were considered poor, fair, good and excellent, respectively [31]. Analyses were conducted on the total sample and separately for the patient groups of MS and psoriasis. As analyses revealed no systematic differences in vignette ratings between patient groups, results are presented with regard to the total sample. Statistical analyses were performed with IBM SPSS V25.

## Quantitative analysis: results

### Participant characteristics

We recruited 50 patients with MS and 50 patients with psoriasis. The mean age was 46.73 (± 14.63) years, 52 participants were female. Sociodemographic characteristics of both patient groups were relatively similar except for gender and educational level. The MS group contained more female (χ^2^(1) = 10.26, *p* < 0.001) and more highly educated participants (χ^2^(2) = 12.82, *p* = 0.002). At baseline, participants’ HRQoL was more negative than the U.S. general population norm of 50 for MCS and PCS. Descriptively, PCS was more positive in patients with psoriasis (45.13 ± 11.33) than in patients with MS (42.68 ± 10.77), while MCS was similar (MS: 45.48 ± 11.68, psoriasis: 45.77 ± 13.18) (Table 1).

**Table 1:** Sociodemographic characteristics of the study participants at baseline (patient questionnaire)

		Patients with MS (*n* = 50)	Patients with psoriasis (*n* = 50)	Total (*n* = 100)
Gender, *n* (%)	Female	34 (68)	18 (36)	52 (52)
	Male	16 (32)	32 (64)	48 (48)
Age in years	Mean ± SD	44.98 ± 13.60	48.48 ± 15.52	46.73 ± 14.63
	Median (range)	43.50 (22–84)	46 (242–83)	44.50 (222–84)
Educational level, *n* (%)	Low	2 (4)	11 (22)	13 (13)
	Medium	16 (32)	23 (46)	39 (39)
	High	32 (64)	16 (32)	48 (48)
Marital status, *n* (%)	Single	19 (38)	21 (42)	40 (40)
	Married/in a relationship	31 (62)	29 (58)	60 (60)
Employment status^a^, *n* (%)	Employed	30 (60)	37 (74)	67 (67)
	In training	3 (6)	4 (8)	7 (7)
	At home/unemployed	8 (16)	5 (10)	13 (13)
	Retired	17 (34)	8 (16)	25 (25)
Living situation, *n* (%)	Alone	13 (26)	11 (22)	24 (24)
	With family/friends/partner	37 (74)	39 (78)	76 (76)
Time since diagnosis in years	Mean ± SD	11.64 ± 9.64	20.88 ± 15.92	16.26 ± 13.90
	Median (range)	10 (1–38)	17 (1–60)	12 (1–60)
Presence of comorbidities, *n* (%)	Yes	30 (60)	32 (64)	62 (62)
	No	20 (40)	18 (36)	38 (38)
SF-12 score at baseline	MCS, mean ± SD	45.48 ± 11.68	45.77 ± 13.18	45.63 ± 12.39
	PCS, mean ± SD	42.68 ± 10.77	45.13 ± 11.33	43.90 ± 11.07

*n* number of patients; *SD* standard deviation; *MCS* mental component summary; *PCS* physical component summary

^a^Multiple responses possible

Of the 100 patients, 93 participated in the follow-up interview. More patients with MS (*n* = 6) than patients with psoriasis (*n* = 1) did not participate in the follow-up; no other differences between these groups were found. Longitudinally, MCS increased (better HRQoL) in both subgroups (MS: + 0.71 ± 9.54, psoriasis: + 3.81 ± 8.19), while PCS decreased (worse HRQoL) in patients with MS (− 1.25 ± 7.04) and increased (better HRQoL) for patients with psoriasis (+ 2.72 ± 8.70).

### Changes in vignette ratings

On domain level, mean changes in vignette rating of the aggregated sample were almost consistently non-significant. According to our assumptions, this indicates that the reference frame of the sample did not change. Furthermore, the change values showed relatively large standard deviations, suggesting substantial variance of ratings over time (Table 2). Subgroup analyses revealed that the relatively large variance of changes in vignette ratings could neither be explained by changes in self-reported HRQoL nor by other sociodemographic factors (data not shown).

**Table 2:** Changes in vignette ratings from *t*1 to *t*2 (*n* = 93)

Anchoring vignette ratings on domain level	Domain score^a^ change		*t*	ICC (95%–CI)
	Mean ± SD	Range (min; max)		
General health I	4.08 ± 24.96^b^	− 60; 60	1.58	0.45 (0.27–0.60)
General health II	2.10 ± 22.08	− 60; 85	0.92	0.39 (0.20–0.55)
Physical functioning I	− 2.42 ± 25.55^b^	− 100; 50	− 0.91	0.40 (0.22–0.56)
Physical functioning II	− 0.27 ± 27.71^b^	− 70; 100	− 0.09	0.22 (0.02–0.41)
Role physical I	7.80 ± 23.81^b^	− 37.5; 100	3.16*	0.22 (0.02–0.41)
Role physical II	1.34 ± 21.37	− 37.5; 100	0.61	0.21 (0.01–0.40)
Role emotional I	4.70 ± 20.51	− 50; 50	2.21*	0.53 (0.37–0.66)
Role emotional II	− 1.61 ± 20.95	− 75; 50	− 0.74	0.38 (0.19–0.54)
Bodily pain I	2.42 ± 19.53	− 50; 50	1.20	0.38 (0.19–0.54)
Bodily pain II	6.45 ± 23.86	− 50; 75	2.61*	0.40 (0.21–0.56)
Mental health I	3.23 ± 17.38^b^	− 37.5; 50	1.79	0.32 (0.13–0.49)
Mental health II	− 0.81 ± 16.46^b^	− 50; 37.5	− 0.47	0.31 (0.11–0.48)
Vitality I	0.00 ± 17.29	− 50; 75	0.00	0.55 (0.39–0.68)
Vitality II	1.61 ± 16.81	− 25; 75	0.93	0.51 (0.34–0.64)
Social functioning I	− 1.08 ± 22.70	− 75; 75	− 0.46	0.11 (− 0.09–0.31)
Social functioning II	2.69 ± 18.96	− 50; 75	1.37	0.41 (0.22–0.56)

*SD* standard deviation; *t t*-test value; *ICC* intra-class correlation coefficient; *CI* confidence interval

^a^Domain scores are calculated by adding up item responses of a domain to a raw scale score and transforming the raw scale score to a 0–100 scale score. Higher scores indicate better HRQoL with respect to the specific domain

^b^Variance of change in self-rating is larger than the variance in change of vignette rating

*p-value < 0.05

At the same time, the within-person agreement of vignette ratings between *t*1 and *t*2 was mainly poor on both domain and single-item level. On the item level, Cohen’s kappa ranged from 0.05 (Vignette I of Item 3a) to 0.38 (Vignette II of Item 6c) (Online Supplementary 2). On the domain level, ICCs of nine vignette ratings was below 0.40 indicating poor agreement. Agreement of the remaining seven ratings was fair (ICC ≤ 0.55) (Table 2). According to our assumptions, this indicates that (test–retest) reliability of the anchoring vignette approach could not be confirmed.

In summary, vignette ratings at *t*1 and *t*2 tended to differ non-directionally, confirming neither reliability nor a directional change in the ratings of anchoring vignettes for the sample and for specific subsamples. These findings give some initial indications regarding questioning the appropriateness of the anchoring vignette approach for investigating response shift in longitudinal HRQoL assessment.

## Qualitative analysis: methods

In order to “disentangle” response shift from actual change in HRQoL, response behavior was analyzed quantitatively (response category chosen) but also qualitatively. We analyzed participants’ verbalized explanation strategies for single vignette ratings. Based on the above mentioned assumptions, we expected participants to provide different explanations at *t*1 and *t*2 in case they changed their vignette rating (choice of a response option). In contrast, we expected participants to provide similar explanations if their rating remained stable. These assumptions were explored in a qualitative manner. We focused on the two anchoring vignettes for the domain General Health (Item 1). We randomly selected 20 participants, transcribed the verbalized explanations at *t*1 and at *t*2 and explored whether explanations remained stable over time. Three researchers independently evaluated the pairs of explanations and judged them as *equivalent, non-equivalent* or *unclear.* The researchers’ appraisals were subsequently discussed in a consensus meeting. Pursuing an explorative approach, we did not predefine specific criteria for reasoning equivalence.

## Qualitative analysis: results

The analysis of participants’ verbalized explanation strategies at *t*1 and *t*2 revealed difficulties, further questioning the appropriateness of the anchoring vignette approach. In the consensus meeting, it was not possible to define unambiguous criteria for determining equivalence. In the following section, reasons for these difficulties shall be illustrated (see Table 3 for examples of verbalized explanations).

**Table 3:**
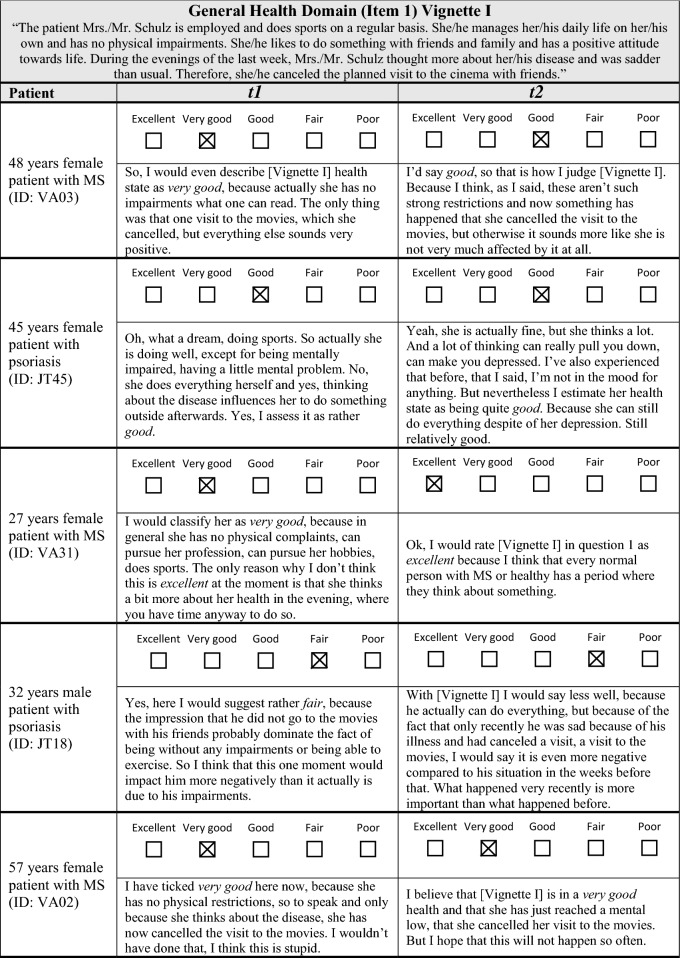

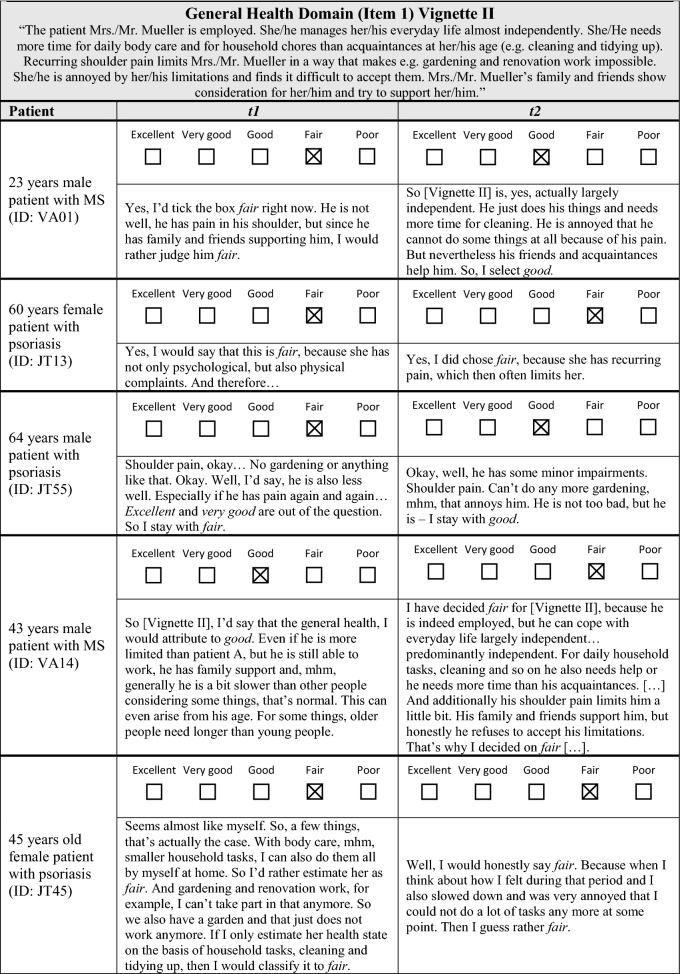
Comparison of verbalized explanation strategies at* t*1 and* t*2

Firstly, the anchoring vignettes contained examples of everyday situations. When explaining their rating, some participants referred to one of these examples at *t*1 and to another – or to more of them – at *t*2 (e.g., Table 3, Vignette I, ID: JT45). From the perspective of the consensus group, it could not be decided whether this indicated a true change in the explanation strategy or rather reflected a random choice among the examples described in the anchoring vignette.

Secondly, some participants described single aspects within the anchoring vignettes in detail at one time point while mentioning it only briefly at the other time point (Vignette II, ID: VA14). Also, some participants changed the order in which they reported on single aspects of the anchoring vignettes. Using the given information, the consensus group members could not decide if these were clear indicators for differences in the valuation of particular aspects which in turn reflect differences in the explanation strategies.

Thirdly, determining a clear threshold that indicates a substantial change in the interpretation and valuation of stated impairments was impossible. In the context of the overall vignette description, it is, for example, difficult to determine whether the statements “she has no impairments” and “these aren’t such strong restrictions” are systematically different or not (Vignette I, ID: VA03). Similar challenges existed when participants at one time point compared HRQoL of a hypothetical patient to their own HRQoL (Vignette I, ID: JT45), to another hypothetical patient (Vignette II, ID: VA14) or to an external person (Vignette I, ID: VA31) while not making this comparison at the other point in time.

All these above mentioned examples emphasize that verbalized explanations may not fully reflect the underlying decision process. The “true” decision process might be in parts unconscious and thus difficult to verbalize comprehensively. Participants seem to randomly select explanations from a variety of different explanations incorporated in the “true” and partly unconscious decision process. Consequently, a presumed change in the verbalized explanation does not automatically mean that the decision process and thus the reference frame changed over time. Therefore, it was agreed that no clear and reliable criteria for equivalence or non-equivalence can be defined because there is a high degree of uncertainty as to whether the verbalizations represent underlying differences in reasoning and reference frame or not.

## Discussion

This was one of the first studies using anchoring vignettes in a longitudinal study in order to explore response shift. As we faced several challenges that led us to question the reliability of the anchoring vignette approach in this context, we shifted the focus towards describing challenges and lessons learned from using anchoring vignettes.

The thorough development of anchoring vignettes initially appeared promising. Pretests revealed that important requirements for subsequent interpretation of vignette ratings, i.e., response consistency and vignette equivalence, were mainly achieved. However, a limitation at this stage was that requirements were only checked in qualitative interviews and based on a small sample. In addition, it became apparent that full achievement of response consistency and vignette equivalence is difficult because both requirements to some degree trade-off against each other. As also indicated by previous research results [14, 32], achieving vignette equivalence requires clear information on hypothetical patients so that participants will understand the descriptions in the same way. Such detailed descriptions, however, may hamper empathy of participants towards hypothetical individuals as they might differ significantly from their own characteristics [33]. Lack of empathy could lead patients to use response categories for vignette rating in different ways than for their self-rating which would then affect response consistency. Although qualitative analysis indicates an overall appropriate balance between both requirements, it cannot be fully proven whether this is sufficient for the interpretation of vignette ratings.

In the subsequent exploratory mixed-methods study, we found that ratings of identical anchoring vignettes fluctuated non-directionally over time. Many participants changed their vignette ratings from *t*1 to *t*2 but positive and negative changes canceled out one another on the group level resulting in non-significant *t*-tests. The changes remained non-significant for different subgroups and variances in changes of ratings could not be explained by specific patient characteristics Thus, we could neither confirm that a directional change in ratings occurred nor that vignette ratings were stable over time (an indicator of test–retest reliability). Consequently, non-directional changes in vignette ratings may occur at random or may be caused by other confounding factors threatening the reliability of the anchoring vignette approach. The level of concentration or distracting thoughts same as learning effects caused by repeated rating of anchoring vignettes may influence the assessment and need to be addressed in future research to judge the conclusive value of the anchoring vignette approach. At this point, we need to emphasize that the current exploratory study was not primarily designed to detect subgroup differences and potential subgroup effects should not be neglected in future research.

We complemented the quantitative vignette ratings by patients’ qualitative explanations for their ratings using the think-aloud method. In doing this, we aimed to gain an in-depth understanding of the complex decision process when answering questionnaires on HRQoL. Although all participants were able to verbally explain their vignette ratings, it remains unclear whether they fully reported the underlying decision process. Accordingly, it is not clear whether and to which extent unconscious processes impacted the decisions. In previous research, the general uncertainty about the completeness and accuracy of information has already been identified as a limitation of the think-aloud method [34]. These uncertainties also hindered the consensus group in defining unambiguous criteria for explanation equivalence and to decide whether individual reference frames changed.

By conducting this study, we aimed at exploring the response shift phenomenon from a different angle. While thus far it has mainly been approached statistically [5, 35], we chose a mixed-methods approach to specifically account for the subjective nature of the target construct itself and the decision process. In particular, the non-directional fluctuation of vignette ratings as well as difficulties in interpreting qualitative explanations indicated that the use of anchoring vignettes might not be appropriate to explore response shift. At this point it must be noted that present results are based on an exploratory study with a non-representative sample of patients with psoriasis or MS and a single generic HRQoL questionnaire only. Generalizability and transferability to other patient groups and other HRQoL questionnaires is therefore limited.

## Conclusion

Although we could not reach the goal of analyzing response shift in the assessment of HRQoL, this study provides profound insight into the use of anchoring vignettes in longitudinal studies and its limitations. Based on the critical results of this study, the anchoring vignette method should be considered with caution at this point in time.

## Electronic supplementary material

Below is the link to the electronic supplementary material.

Supplementary file1 (DOCX 17 kb)

Supplementary file2 (DOCX 737 kb)
