# Supplementary material for: Challenges and lessons learned from using anchoring vignettes to explore quality of life response behavior
Source: Qual Life Res. 2020 Apr 18;29(8):2149–59. doi: 10.1007/s11136-020-02488-4 (PMC7363714; doi:10.1007/s11136-020-02488-4)
Supplement: Supplementary file 1 — Supplementary file1 (DOCX 17 kb) [file 11136_2020_2488_MOESM1_ESM.docx]

**Online Supplementary 1** Anchoring vignettes translated into English language.

The following 16 anchoring vignettes were assessed with regard to the items of the SF-12 questionnaire. In detail, each anchoring vignette contained statements related to one of the eight domains of the SF-12 questionnaires and was assessed using the items related to this specific domain of the SF-12. Anchoring vignettes were available in male and female version to match the patient’s gender. Only the female version is presented here.

| **General Health I**  The patient Mrs. Schulz is employed and does sports on a regular basis. She manages her daily life on her own and has no physical impairments. She likes to do something with friends and family and has a positive attitude on life. During the evenings of the last week, Mrs. Schulz thought more about her disease and was sadder than usual. Therefore, she canceled a planned visit to the cinema with friends. |
| --- |
| **General Health II**  The patient Mrs. Mueller is employed. She manages her everyday life almost independently. She needs more time for daily body care and for household chores than people at her age (e.g., cleaning and tidying up). Recurring shoulder pain limits Mrs. Mueller in a way that makes for example gardening and renovation work impossible. She is annoyed by her limitations and finds it difficult to accept them. Mrs. Mueller’s family and friends show consideration for her and try to support her. |
| **Physical Functioning I**  The patient Mrs. Weber takes care of the household on her own. Cleaning, tidying up and cooking do not cause her any problems. When working in the garden or carrying heavy objects, family and friends support her. She reaches her apartment on the third floor without any problems. However, she finds it exhausting to carry heavy shopping bags to the third floor. |
| **Physical Functioning II**  The patient Mrs. Krueger does household chores like cooking and cleaning on her own but requires twice as much time for them as friends at her/his age. When cleaning windows or carrying out repairs, she is dependent on the support of her partner. Due to knee pain, she had to give up on her hobby, bowling, about a year ago. Mrs. Krueger also has pain in her knee when climbing stairs and tries to relieve her knee by using the railings for support. If possible, she uses an elevator. |
| **Role Physical I**  The patient Mrs. Koenig usually rides her bicycle to work (3 km). Due to knee pain, she did not cycle to work for 3 consecutive days in the last 4 weeks but took the bus instead. The pain did not affect her during work. However, after work, Mrs. Koenig did not take care of the household as planned during these 3 days because of the knee pain. |
| **Role Physical II**  Endurance and strength of Mrs. Winter are limited. Therefore, she avoided going to the supermarket (1 km) for several weeks. Her partner does the shopping for her. Due to the physical constraints, she has been on sick leave for 1 week in the last 4 weeks. During this time, she did not take care of the household. In her office job, Mrs. Winter mainly worked on tasks that needed to be completed urgently but postponed other tasks to a later date. |
| **Role Emotional I**  Thoughts about her disease made Mrs. Wagner sad on four days in the last four weeks. During work (full-time job), she was able to suppress her dejection well and did her job as usual. After work she lacked motivation on those four days and only half-heartedly completed tasks such as shopping, doing the laundry and tidying up. Mrs. Wagner’s partner supports her at times, when she is sad and crestfallen and manages to motivate her to take part in activities and arrange meetings with friends. |
| **Role Emotional II**  The patient Mrs. Neumann is annoyed by the fact that recently, she can no longer keep up with the tasks at work due to her illness. She cannot concentrate well and is distracted. In the past 4 weeks this also affected her everyday life at home. In the evenings, she was listless and postponed household chores such as shopping, washing up and doing the laundry to the weekend. On weekends, she did these tasks quickly and superficially to have more time to recover from the work week. |
| **Bodily Pain I**  The patient Mrs. Meier has had headaches on two consecutive days in the last four weeks. On both days she took painkillers to be able to go to work. The remaining time she was free of pain. |
| **Bodily Pain II**  The patient Mrs. Lange has pain in her knee, especially during joint loading (e.g. climbing stairs, exercising). On 5 days in the last 4 weeks, the pain was so severe that she could not go to work and was certified sick by her physician. |
| **Mental Health I**  The patient Mrs. Klein finds it difficult to accept that she is no longer as physically active as she was a few months ago. In 2-3 situations in the last 4 weeks, she was sad when she realized that she could not keep up physically with friends at her age. On the other hand, Mrs. Klein is happy to have friends and family who support her when necessary and take care of her health. |
| **Mental Health II**  During the last 4 weeks, the patient Mrs. Zimmermann became aware of her physical limitations due to the disease several times a week. It makes her sad when she notices that she cannot play with her children, cannot and does not want to go swimming and cannot keep up with the others at work. In addition, it makes her feel unhappy and helpless that the new medicine does not have the desired effect. |
| **Vitality I**  Usually, the patient Mrs. Hofmann is looking forward to the challenges of the day and describes herself as a fighter type. On about 3 days in the last 4 weeks, she was depressed and sad. On these days, she would have preferred to retreat from public life. |
| **Vitality II**  Mrs. Weiss feels tired and exhausted since she had stopped exercising a few months ago due to her illness. In the last 4 weeks she was only once motivated to do something with friends. She prefers to spend her time reading or watching TV. |
| **Social Functioning I**  The patient Mrs. Braun finds it difficult when many people speak at the same time at regular tables or parties. In these situations, she has to concentrate a lot in order to be able to follow conversations. For this reason, she cancelled an invitation to a friend's birthday party in the past 4 weeks. On the other hand, she is looking forward to activities with her family. She is also happy to meet with individual friends. |
| **Social Functioning II**  The patient Mrs. Schroeder feels increasingly uncomfortable in public. She avoids visits to restaurants or swimming pools because she feels that other people are constantly watching her. Since Mrs. Schroeder cancelled two planned activities with her partner in the last 4 weeks, more and more conflicts have arisen. |
