# Supplementary material for: Challenges and lessons learned from using anchoring vignettes to explore quality of life response behavior
Source: Qual Life Res. 2020 Apr 18;29(8):2149–59. doi: 10.1007/s11136-020-02488-4 (PMC7363714; doi:10.1007/s11136-020-02488-4)
Supplement: Supplementary file 2 — Supplementary file2 (DOCX 737 kb) [file 11136_2020_2488_MOESM2_ESM.docx]

**Supplementary Material 2** Graphics on changes in anchoring vignette ratings.

Changes in vignette ratings from *t*1 to *t*2 displayed on single item level. The width of the lines represents the proportion of participants who gave the respective anwer. Cohen’s kappa (κ) and the proportion of participants who did not change their vignette rating from *t*1 to *t*2 are given to the right of each graph.


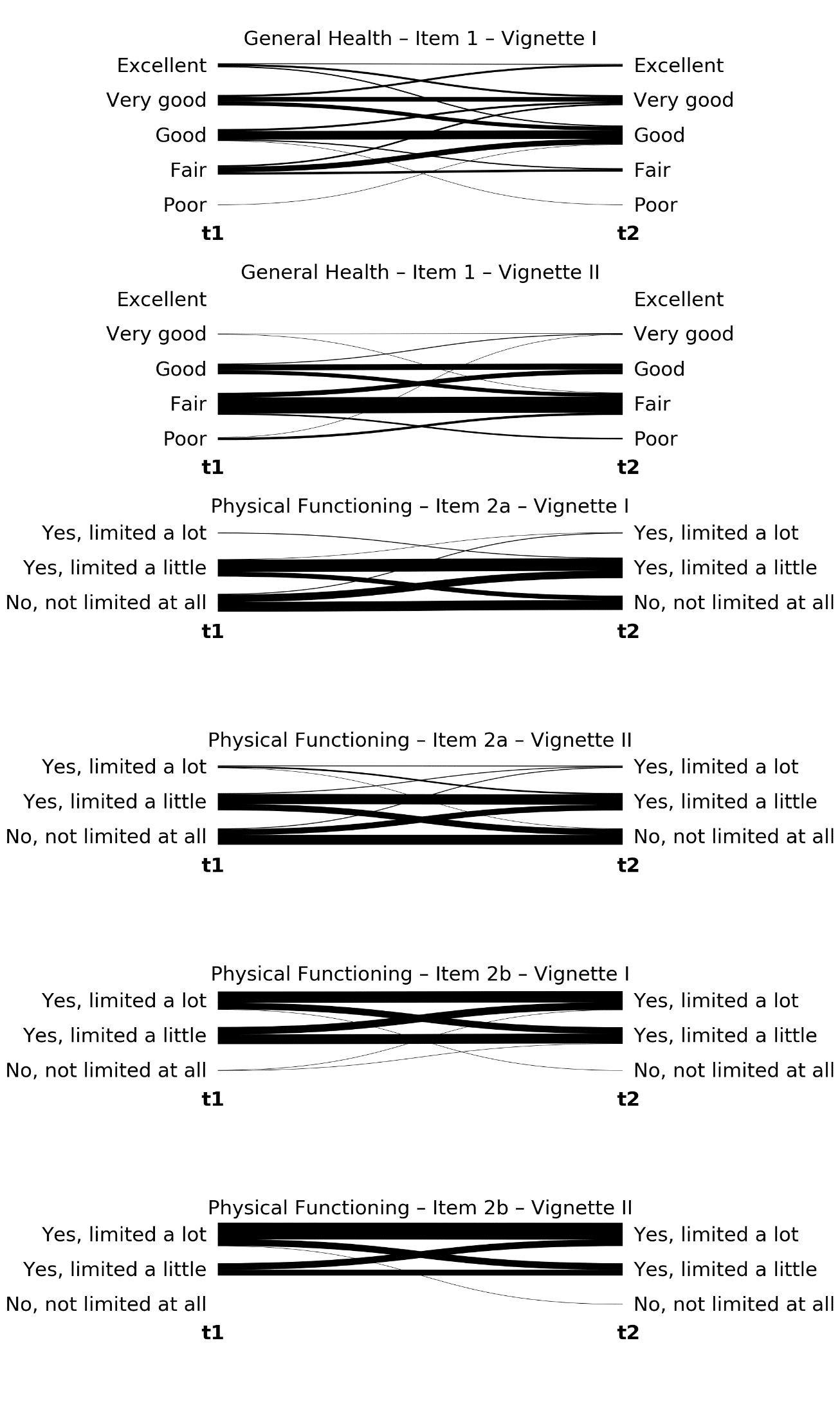


κ = 0.22

no change: 45.2 %

κ = 0.27

no change: 61.3%

κ = 0.27

no change: 61.3%

κ = 0.19

no change: 58.1%

κ = 0.18

no change: 62.4%

κ = 0.24

no change: 57.0%


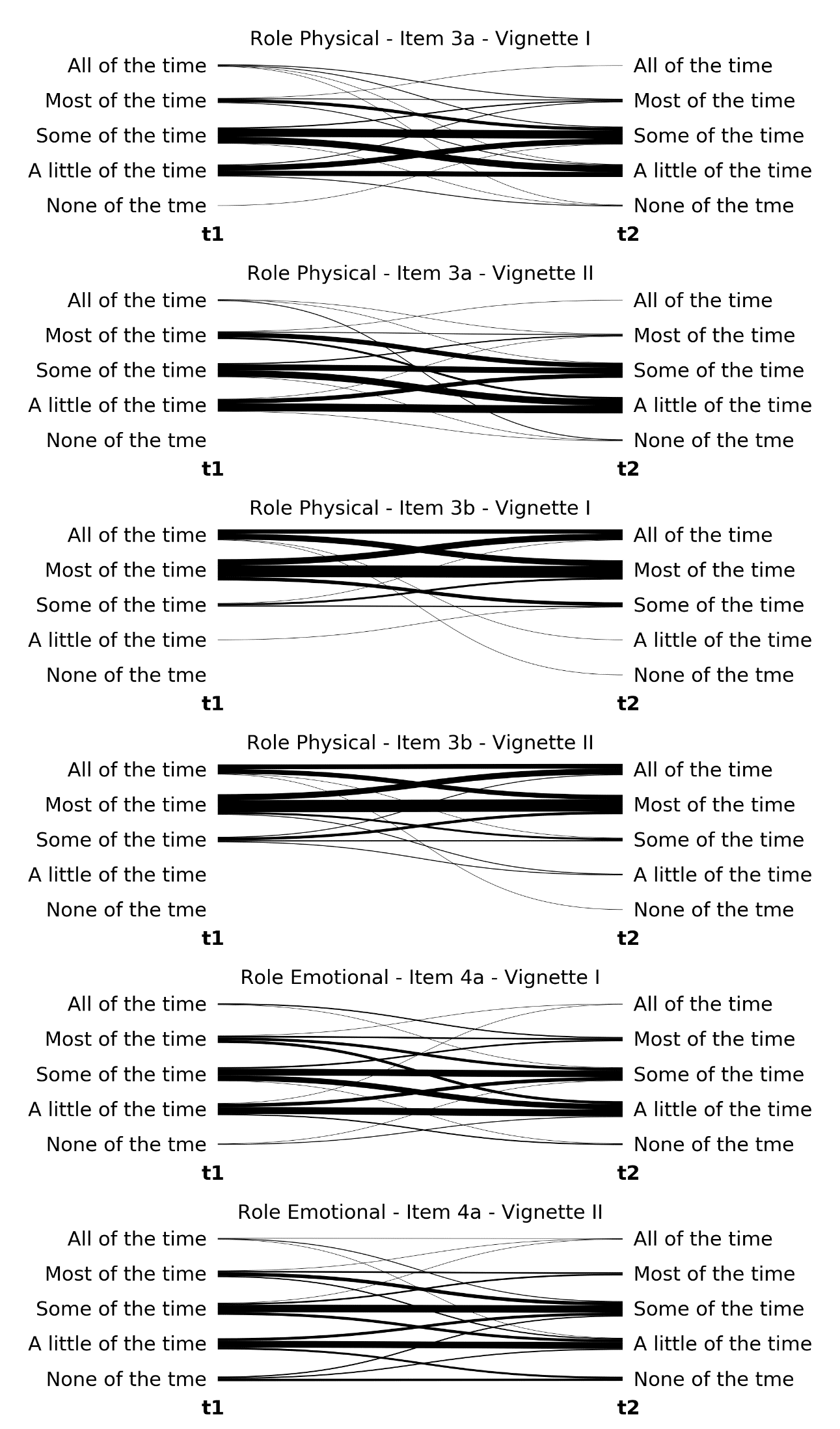

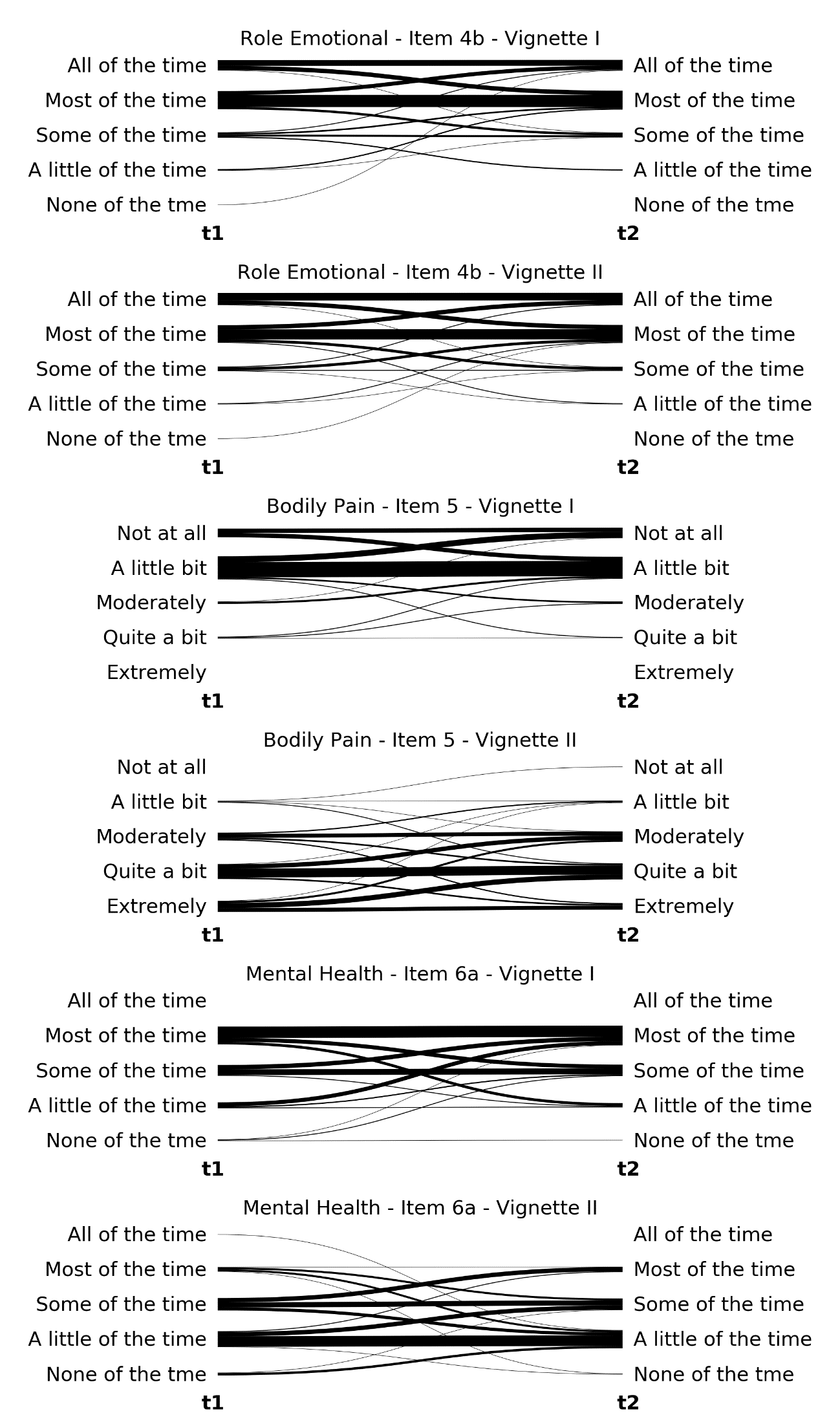


κ = 0.28

no change: 54.8%

κ = 0.15

no change: 41.9%

κ = 0.15

no change: 75.3%

κ = 0.09

no change: 39.8%

κ = 0.05

no change: 38.7%

κ = 0.09

no change: 47.3%

κ = 0.19

no change: 50.5%

κ = 0.31

no change: 50.5%

κ = 0.20

no change: 50.5%

κ = 0.15

no change: 54.8%

κ = 0.23

no change: 47.3%

κ = 0.13

no change: 44.1%


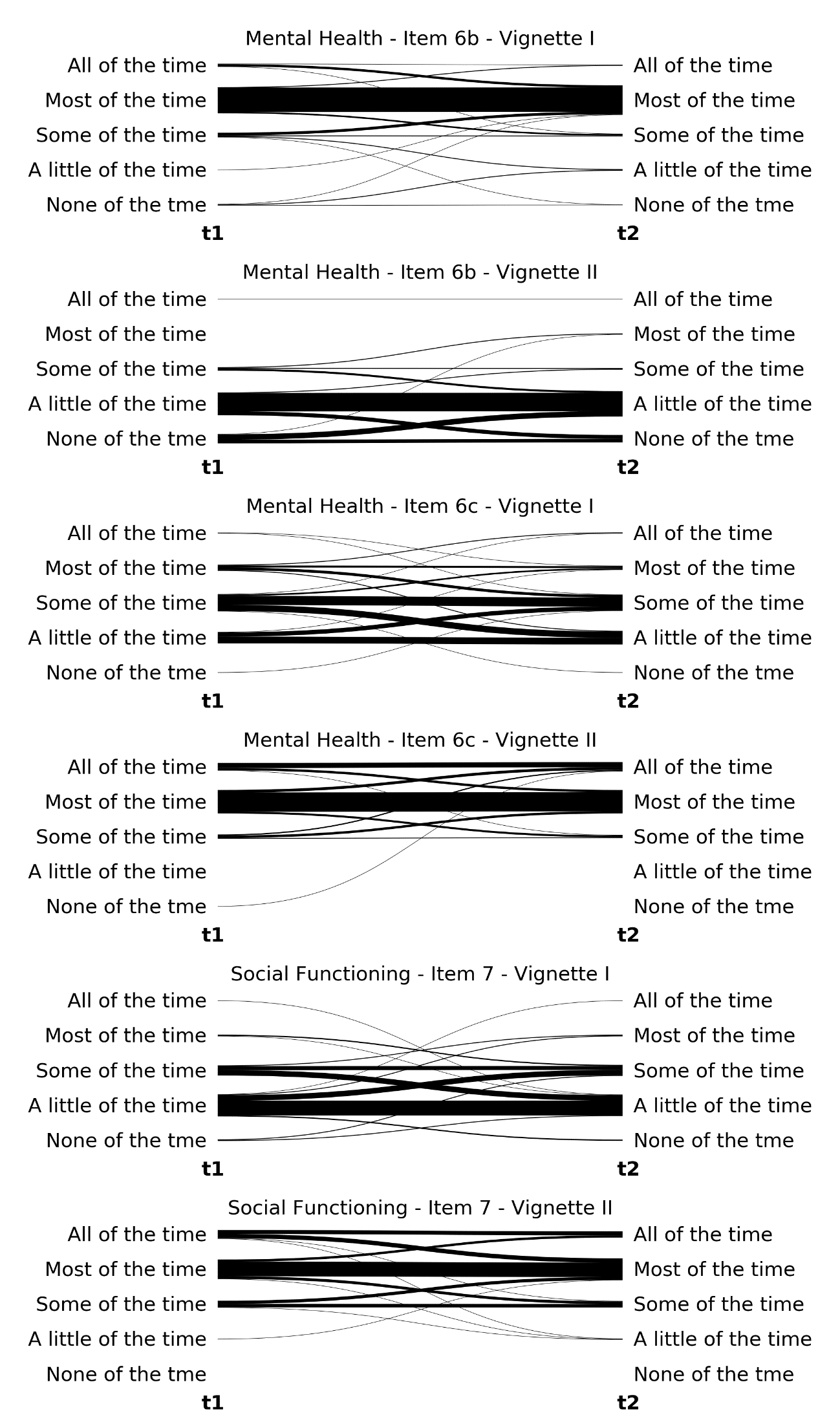


κ = 0.09

no change: 51.6%

κ = 0.32

no change: 60.2%

κ = 0.38

no change: 68.8%

κ = 0.19

no change: 48.4%

κ = 0.26

no change: 63.4%

κ = 0.24

no change: 71.0%
